# Atlanta Academy of Medicine

**Published:** 1874-06

**Authors:** 


### Atlanta Academy of Medicine.

JAMES B. BAIRD, M.D., Reporter.

Atlanta, April 13, 1874.

Dr. W. S. Armstrong, President, presiding.

Dr. Battey continued the report of a case first brought to the attention of the Academy on the 26th of last January. (See proceedings Atlanta Academy of Medicine, in March number, for detailed history of case.) A lady, æt. about thirty, laboring under a supposed uterine malady of some year's standing, applied to him in September last. There was decided hypertrophic elongation of the cervix uteri; the os protruding between the labia was kept in a constant state of irritation by the pudendal hairs and the clothing. Endo-metritis existed, but no prolapsus. In douglas' cul-de-sac, on left side, discovered a tumor about the size of a black-walnut; freely movable; fluctuating. A smaller tumor, in a corresponding situation on the right side, probably an enlarged and swolen ovary. The patient was seen from month to month, and the tumor on the left side observed to gradually increase, until it had attained to the size of a small orange. Two weeks ago, March 30, he operated, assisted by Drs. J. P. Logan, W. F. Westmoreland, J. G. Pettus and W. S. Kendrick. The patient was etherized; placed upon left side, semi-prone posture;

Sims' speculum introduced. With a pair of strong scissors an incision was made into douglas' fossa, in the median line, behind the uterus, care being observed to avoid wounding the rectum. The tumor was seized through the incision with a pair of forceps, punctured, and two fluid ounces of a thick, viscid, oleaginous fluid allowed to escape. He then observed hairs protruding through the puncture, and at once recognized the growth to be a histoid cyst of the ovary. It was withdrawn with facility into the vagina. In deference to the opinion of the medical gentlemen present, though in opposition to his own judgment in such cases, a ligature was thrown around the pedicle, and the tumor removed. A loop of small intestine and the right ovary followed the tumor through the incision. These were returned. The ovary was healthy. The wound was brought together by a single point of suture in the center. During the afternoon and early part of the night she suffered extremely; great pain at the seat of the operaion; just what he had expected; due to the ligature; experienced the same difficulty in the use of the ligature in an ovariotomy done in Rome some time ago. Pulse went up to ninety per minute; an opiate was given. He feared trouble, but was, however, gratified at the early disappearance of the unfavorable symptoms. In thirty-six hours the pulse was seventy-two; in sixty hours, sixty-six; not higher since; after the first twelve hours but little pain; progressed very comfortably. On the fourth day introduced speculum and examined wound; suture removed; healing gone on well, and union by first intention has been secured, so far as he can judge by the touch, except at the point at which the ligature attached to the pedicle passes out. The tumor was presented for inspection. The cyst wall was thick and resisting, having the appearance of skin inverted, so that the epidermis formed the lining membrane of the cyst. Besides the fluid already described, it contained a ball of long, reddish colored, loose hair; also hairs growing from the interior, and a bone imbedded in the tissues about half inch in length and three lines in thickness.

Dr. B. remarked that he was gratified to be able to present this specimen to the Academy, and would make it the text of what he had promised to say about histoid tumors at a future time. Here we have not a fœtus degenerating, and finally disappearing, a few of the tissues remaining, but a tumor, progressing and growing at an increased ratio. The hair in its interior is not that of a child, and imbedded in its walls is an irregular osseous growth, not going backwards, but also progressing. So far as he is informed this is the third time this operation of *vaginal ovariotomy* has been performed. The first by Dr. T. Gaillard Thomas of New York, the second by Dr. J. T. Gilmore of Mobile.

Dr. Battey also reported another case of ovariotomy done in Crawford county, on Saturday last, 11th instant. A lady, æt. 43; six children, youngest æt. 8. Six years ago she first noticed a tumor in the left iliac fossa, easily movable; some pain. Six or eight months subsequently a similar tumor was observed in the right iliac fossa, also freely movable, and giving slight pain. These tumors were examined by Drs. Searcy and K. P. Moore of Crawford county, who confirmed the statements of the patient as to the early location of the tumors. These tumors continued to grow until they seemed to coalesce and form one tumor, with a deep sulcus between. The tumor on the right seemed to outgrow the original tumor on the left. The enlargement of the abdomen was much greater than in an advanced stage of gestation. The patient was anæmic and considerably emaciated. She presented the condition described by Spencer Wells as the ovarian cachexia, which requires nice discrimination to distinguish from cancerous cachexia, which it somewhat resembles. The tumor presented a smooth, regular contour, not lobulated. On account of the great distension of the abdominal walls, the existence of fluctuation could not be positively determined. The moral of the patient was fine; she was anxious for the operation. It had been deferred until she saw death immediately before her. She was etherized, and an incision four and a half inches long made through the abdominal wall in the median line. A month ago he decided that no anterior adhesions existed, and this opinion was confirmed by the facility with which a long probe, passed through the incision, was swept over the entire anterior surface of the tumor, from the pelvis to the ensiform cartilage, and extending laterally as far as the curved abdominal walls would permit. The uterus was drawn up, as is not unfrequently the case if the pedicle be short or the tumor large. From the size of the tumor, he was unable to determine whether or not the uterus was movable, independently of the tumor. The tumor was seized with a vulsellum and a trocar plunged into it. The external envelop was so firm that it was necessary to introduce the trocar with a boring motion, as it resisted direct force. The instrument was carried to a depth of six inches into the tumor, but no fluid escaped. It was then introduced into another part of the tumor, with like result. The incision was then extended above the umbilicus. The capsule of the tumor was divided by a stout pair of scissors, and an attempt to break up the contents decided upon, as it would have been impossible to turn out the tumor entire. He found, upon passing his hand into the sac, an aggregation of solid masses incased in the capsule, and separated by membranous partitions, varying in weight from one ounce to, perhaps, six or eight pounds. The number could hardly be estimated. After removing as many as possible from the interior, the capsule was seized with a vulsellum and drawn out. Twelve inches of adherent small intestine was withdrawn with it. This was separated without injury. The ureter of the left side was imbedded in it; this was dissected out. The tumor was adherent to the descending colon, and very close adhesions existed between it and the uterus, so that it was impossible to separate them, though the contour of the uterus could be readily made out. The pedicle was incised above the uterus, and fortunately, the uterus having been so long elevated, he was enabled to treat the stump outside the abdomen without any strain upon it. The amount of blood lost during the operation was small, less than is usual in amputation of a limb.

Evidences of decided shock became apparent during the latter stages of the operation. The pulse began to flag, and the operation was hurried up as much as was consistent with its proper performance. All was satisfactory so far, but after dressing and putting her to bed, the indications of collapse continued and increased, and, notwithstanding the free use of brandy, morphine, etc., by the mouth and rectum, with heat and frictions, she succumbed one hour after the completion of the operation. No vessels were found in the center of the stump, which was white and fibrous. It was allowed to lie in the lower angle of the wound. The fatal issue was palpably due to shock.

A specimen of the tumor was presented for inspection, and Dr. B. remarked that it was a fibroma of the ovary, a rather rare form of disease. It is not a fibroid tumor of the uterus. Was astonished at its size; it would have reached three pecks of solid material, had the tumors been measured as turnips, and its weight approximated thirty-five pounds, as determined by Drs. Rudicil and Kendrick. A chain of little cystic tumors ran up along the side of the bladder. After determining the character of the tumor, there was nothing hopeful in sewing the abdomen up; was there in removing the tumor? Spencer Wells, Atlee and others have recorded sucessful cases apparently as bad as this.

Thinks it would have been very bad practice to have closed the wound without removing the tumor, considering the facility with which it was removed.

Dr. Taliaferro inquired if there was any distinct mark of separation between the tumor and the uterus.

Dr. Battey replied that the touch easily made out the distinct form of the uterus. This tumor was very elastic and much less firm than fibroids of the uterus. The pedicle was attached to the whole fundus and side of the uterus.

Dr. W. F. Westmoreland observed that he felt special interest in the details of this subject, as he had set apart this week for removing an ovarian tumor. He diagnoses no attachments, tumor freely movable, no pain. If, after an exploratory incision, attachments are found, shall the operation be proceeded with, or the wound closed and the tumor allowed to remain?

Dr. Battey replied that there was a rule, well defined. When the surgeon has opened the abdomen, whether the ovarian tumor be solid or cystic, if it is so attached that he thinks the viscera will be seriously damaged by its removal, close up the wound. If he decides that its removal will not prove detrimental to the viscera, go on. If it is not a rule, it is at least the practice of good operators not to be deterred from removing the tumor if it prove to be malignant. A considerable number of years have elapsed in such cases without a return of the tumor; besides, malignant disease of the ovaries, as now understood, is not so apt to return as malignant disease of the breasts. In answer to an inquiry, he added that there were no means known by which the existence or non-existence of posterior attachments of ovarian tumors could be positively determined.

Dr. W. F. Westmoreland asked if there were any special signs of malignancy.

Dr. Battey—Yes; lancinating pain, a "hob-nailed" surface of the tumor, etc. So far as he is informed, malignant tumors of the ovary are never smooth and regular in their contour. Attachments are not more frequent than in benign tumors. He referred to a case met with some years ago. The abdomen was opened. The tumor presented the "hob-nailed" surface, and the abdominal wall was studded with small tumors. In view of its malignant nature the wound was closed. The patient made a rapid recovery from the exploration, and survived five months. If he should meet with a similar case to-day, would remove the tumor.

Dr. Armstrong said if the tumor was small, posterior attachments might be made out; but if large it was impossible.

Dr. W. F. Westmoreland inquired if, after the operation, the stump could not be dressed through douglas' cul-de-sac, or the ligatures brought through into the vagina.

Dr. B. replied that the ligatures had been thus disposed of in at least two instances; one by a German operator, one by an English surgeon.

Dr. DuPre discouraged the operation if the attachments were extensive, and particularly if adhesions existed between the tumor and the mesentery, as the division of such vascular tissues, apart from the oozing of blood, established many points of inflammation.

Dr. Westmoreland thought more was dependent upon the character than the extent of the adhesions. The danger is increased in proportion to the amount of the dissection.

Dr. Battey expressed the opinion that, notwithstanding the existence of extensive adhesions, if the tumor could be removed without interfering with the structure or the physiological functions of the organs, the operation should be proceeded with.

Dr. R. B. Anderson, of Roswell, reported several cases of puerperal convulsions, and asked the opinion of the Academy as to the cause and the best plan of combating this terrible affection.

Several members responded to his inquiry.

Adjourned.

---

A. W. CALHOUN, M.D., REPORTER.

---

ATLANTA, April 20, 1874.

The President, Dr. Armstrong, in the chair.

Dr. Battey read a number of cases from Spencer Wells on Ovarian Tumors, and one from a late Journal, in all of which very formidable adhesions were encountered and successfully overcome, in support of his assertion that extensive adhesions *alone* did not contra-indicate ovariotomy. Called attention to the fact that of the thirteen cases cited from Mr. Wells of this character, no less than twelve made good and perfect recovery, and the remaining case survived the operation sixty hours. He remarked that these appeared, from Mr. Wells' record, to be the very cases that did well. He likewise read the authority of Prof. Peaslee upon this point.

Dr. Armstrong asked if Spencer Wells reported any cases in which he declined to operate at all.

Dr. Battey replied, yes, a few. Mr. Wells has not been in the habit of singling out only the favorable cases. He operates on all as they come, where there is any ground for hopeful issue.

Dr. W. F. Westmoreland thought that each case of adhesions should receive separate consideration, and, so to speak, should govern itself. Some are easily broken down, and are of little consequence, while others are quite serious. Each case should rest upon its own merits.

Dr. Logan thought it highly desirable that the operation should be completed when once begun, even though adhesions did exist, provided any reasonable hope was left of cure.

Dr. Battey said the rule which governs ovariotomists, as laid down by Peaslee, is that no amount or kind of adhesions, discoverable before the operation, contra-indicates ovariotomy; "though, if extensive and vascular, or pelvic adhesions be discovered on opening the abdomen, they may in very rare instances justify its abandonment. The presumption, therefore, should be that no case is to be abandoned on account of adhesions merely, if possible to overcome them; and the operation should actually be left unfinished only when they clearly necessitate such a prolongation of the operation, or such a loss of blood, or both, as will probably prove fatal in the patients actual condition." Ovariotomy is a dernier resort; it is a struggle for life in the face of a certainly impending death. This is to be considered, and hence a surgeon is not to be justified, when once having opened the abdomen, in denying his patient the chance of life by timidly shrinking from the encounter with adhesions. It is his bounden duty to go boldly forward in dealing with adhesions, provided no unreasonable violence be done to vital organs.

Dr. Armstrong thought there must be something in each case to guide us. There are doubtless some cases where it would be much safer to abandon the operation before its completion.

Dr. W. F. Westmoreland exhibited a fatty tumor weighing six pounds, taken from the arm of a lady twenty-six years old. It was situated at the junction of the upper and middle third of the arm, on the anterior and inner aspects. A mixture of chloroform and ether was employed as the anæsthetic.

Dr. Logan desired to know if there was any special advantage to be derived from such a mixture.

Dr. Armstrong and Dr. W. F. Westmoreland thought the mixture had the disadvantages of each of its constituents.

Dr. Battey thought there were disadvantages arising from the great difference in specific gravity of the two fluids, the chloroform being very much heavier than the ether, tends towards the bottom in the mixture. The ether, being far more volatile, evaporates more rapidly and leaves behind a constantly increasing proportion of the chloroform. The chloroform is inhaled last, at the time of greatest danger, and when its intensifying powers are less needed than in the earlier stages. He gives them separately, pushing the ether steadily, and employing a little chloroform from time to time, as needed, to intensify the effect and assist the ether in bringing the patient more rapidly under its influence. Where the lungs are filled to an alarming extent with the vapor of chloroform, he turns the head downwards, and allows the vapor to flow out of the lungs, which it will readily do, by reason of its great density.

Dr. Armstrong reported the case of a lady who had aborted, and when seen by him had but a slight "show." He advised her to remain quiet. Saw her again two days afterwards, and found her suffering severe pain and some discharge. He tamponed the vagina, and advised quiet. A few days after this found a soft mass in the uterus, but it could not be removed. When he reported the case to a previous meeting, it was advised by some of the members to remove the mass, whilst others thought it should be let alone, and it would probably come away of its own accord.

Dr. W. F. Westmoreland had a case where a three months fœtus was passed off, but no after birth. Upon examination, a few days since, found the placenta still remaining. He wished to hear the views of the Academy as to whether it should be removed or let alone. There is no hemorrhage, nor any pain.

Dr. Taliaferro thought it best to remove the placenta by means of cloth, or other kind of tents, in order to prevent decomposition taking place. A retained ovum and retained placenta are quite different things. In the case of Dr. Westmoreland, he thought the placenta should by all means be removed by the use of tents or otherwise.

Dr. Battey was of the opinion that she should be put to bed and left at rest. Would not even give ergot, if she is now doing well. Under the circumstances would simply keep her quiet, and feel no uneasiness so long as she is doing as well as now.

Dr. Baird inquired if it was not the duty of the physician to feel uneasiness so long as the placenta remained.

Dr. Tailiaferro contended that by timely removal of a retained placenta many of the numerous evils which sometimes follow this condition of things, would be effectually prevented.

Dr. Battey, in reply to Dr. Baird's question, answered: No, unless some symptoms call for uneasiness. The sick need a physician, not they that are well.

Dr. Logan remarked that after a separation between the ovum and the uterus had occurred, it was the rule to get rid of the uterine contents. After the expulsion of the ovum the great danger of hemorrhage is past. Thinks there is no doubt about the use of the tampon, where there is hemorrhage, both as a means of checking this, and also of dilating the uterus, and thus facilitating the expulsion of its contents. The tampon not only checks hemorrhage, but is also an excellent means of exciting contraction of the uterus. It is always advisable to remove any portion of the ovum or placenta which may be remaining, and which may become decomposed, and hence injurious.

Dr. J. G. Westmoreland was of opinion that more harm would result from the attempted removal of the contents of the uterus than by letting the organ have its own way. He had never seen any evil result from leaving it to itself. The uterus usually empties itself, at the latest within seven or eight days, according to his observation.

Dr. Cumming would not take any risk, but would use the tampon at once. The pressure of the tampon upon the mouth of the uterus causes the blood to coagulate, and becoming adherent to the tampon when the latter is removed, as it should be every day, the contents of the uterus would ultimately come away at the same time. He would not, by any means, use ergot.

Dr. Logan thought the tampon *the* remedy in cases of abortion. Put it there and let it alone.

Dr. Battey did not regard it a matter of indifference whether we tampon a patient or not. Tamponing ought not to be esteemed a trivial matter. It is a "nasty thing," shocking to the delicacy of a sensitive lady. He always felt that both a good reason and a suitable apology were called for whenever he proposed the remedy. Invaluable, as it certainly was, in suitable cases, it should not be resorted to upon trivial pretexts.

Adjourned.

ATLANTA, April 27, 1874.

The President, Dr. Armstrong, in the chair.

Dr. Battey exhibited a Double Monster, lately received by him from Bartow county, Georgia, with the following history from the attending physician, Dr. R. Webb Willis, of Adairsville:

"I was called to see Mrs. R. at midnight, on the 19th July, 1873. Found her suffering premonitory pains of labor, with slight hemorrhage, but no dilatation of the os. Her abdomen was very large, tense and hard. I asked if she was at full time. She replied no, only seven and a half months. She had borne six children, but stated that her sufferings with them all together did not equal that experienced with this one. Her statement was that she felt as if her stomach and lungs were continually trying to get up into her throat. She was very restless, had intense headache and flushed face. I administered a quarter grain of morphia hypodermically, which quieted her, and ordered acetate of lead and opium for the hemorrhage. Also—

℞.—Chloral Hydrate, . . . . . ʒ ij.

Chloroform, . . . . . . . gtt. xx.

Syr. Zingiberis, . . . . . . ℥ ij.

Aquæ, . . . . . . . . ℥ ij.

M. Sig. Teaspoonful pro-re-nata to quiet the labor pains and secure rest.

"Called on the 20th at 3 P. M. She had experienced relief from the remedies, but still complained of the great pressure and constant desire to draw a long breath.

"Called again on the 22d, and found her sitting up, free of pain, but unrelieved as to the respiration.

"I was summoned at 4 P. M., on the 23d. Upon examination, I found that labor had already commenced, cephalic presentation. I noticed nothing unusual; the two heads seemed as one. I assured the friends that it would be in vain to try to prevent the premature labor. At 8 P. M. I made a second examination, and ruptured the membranes. An immense discharge of waters took place; indeed, so copious as to saturate the bedding, and run off upon the floor half across the room. As nearly as I can estimate, there was twelve or thirteen pints. I now discovered, upon examination, the two heads presented, the one by the vertex and the other partly by the face. Passing my finger between the two heads, I felt what I took to be a shoulder, but could not satisfy myself fully as to the situation. As labor seemed to be progressing favorably, I determined to make no further interference. The labor terminated at 1:30 A. M. The presenting occiput passed under the pubic arch, rotated to the left, and was quickly followed by the other head, in the position as I found them at the second examination, the complex body and limbs following as usual.

"The mother was reared in Baker county, Georgia, is thirty-two years old, in excellent health, weighing about one hundred and fifty pounds. She had previously borne six children, at single births. She has three sisters, all of whom have borne children; no twins in the family. She had a good getting up after the labor."

The specimen exhibited consists of two male children, united by the anterior and upper portions of the trunk. The head and neck of each is perfect and distinct. The union commences at the top of the sternum, and extends down to a point just below the insertion of the common umbilical cord, which contains a double set of vessels. An arm of each presents in front, while in the rear is to be seen a double arm, terminating in two rather imperfectly formed hands. The pelvis of each is distinct, and with the lower extremities normally developed.

Dr. Armstrong read an article descriptive of a case, and his treatment of fractured patella, resulting in bony union.

Dr. Dupre gave the treatment practiced in Nashville for such injuries, viz.: a brass ring strapped down over and around the patella, the fragments having first been brought together by extending the limb, etc. Can call to mind two cases thus treated, with rapid recovery and bony union. The advantage of the metalic ring is that it is a good conductor of heat from the limb. A similar practice was found to have been in use near a century back.

Dr. Battey saw the same in Paris in 1859, and thought it was a French device. Also saw Malgaigne's clamp much in use.

Dr. Calhoun mentioned that the use of this clamp, as originally practiced in Paris, viz.: fastening the teeth into the fragments of the patella through the skin, and screwing them firmly together, had been abandoned in the German hospitals; and that Langenbeck, in Berlin, had made the improvement of fastening strips of gutta percha softened in water above and below the patella, broken into two fragments usually, and into these strips imbedding the teeth of the clamp and screwing the parts together, which was very readily done. This plan but seldom failed to accomplish the happiest results.

Dr. Woodhull, U. S. Army, reported the case, in the practice of a Boston physician, of a young lady who fell down a flight of steps and felt something in the knee give way, but had sufficient presence of mind to remain quiet till assistance came. It was found that the patella was fractured transversely. Used an ordinary bandage and Smith's anterior splint, and suspended the limb, with ice-bags resting on the injured part. The cure was good, with excellent use, but *complete* flexion of the limb was never afterwards practicable, though the use of the limb is otherwise perfect.

Dr. Battey had no doubt but that the "ring treatment" was a hundred years old. In fact, in considering our scientific progress, we are continually reminded of the fact that there's "nothing new under the sun." We have but to turn to any of our very old works and we find clearly pictured many of the ideas and designs that we now look upon as new and original.

Dr. W. F. Westmoreland brought before the Academy four hemorrhoidal tumors, three internal and one external, which he had a few days previously removed. Thought the best mode of removal and the safest, to be by means of the ecraseur chain. The ligature causes intense suffering and sometimes even brings on tetanus. The pain thus produced oftentimes lasts for a week. More patients die from the use of the ligature than from any plan of operating. The great trouble with the wire ecraseur is that the parts are cut through too soon. Hence he prefers the chain. Chassignac not only brought down all the hemorrhoids, but also the neighboring mucous membrane, and applied the ecraseur. But this has a tendency to produce a constriction of the rectum. He has abandoned this mode of treatment entirely. He directs the patient to evacuate the bowels and force down the tumors. Then, after transfixing them with a threaded needle, chloroform is given and the chain ecraseur applied. No blood of consequence was lost in the case from which these tumors were taken.

Dr. Logan testified to the pain following the use of the ligature, but considered it a certain way of dealing with the affection. Has often found so much pain that he thinks it doubtful that he will again resort to this operation. Upon an average, it takes eight or ten days for the tumors to fall off.

Dr. J. G. Westmoreland was sure the ecraseur was by far the best instrument and did not know that the ligature was at all resorted to at this day. The ecraseur is also painful, but it is comparatively momentary. He had a case who walked about the streets three days after the operation.

Dr. Logan did not wish to be understood as making use of an obsolete operation. In all the recent works on surgery, the ligature would be found prominently mentioned. Pain is not invariable after applying the ligature, as instanced in a case recently operated upon by himself. He thought well, however, of the ecraseur,

Dr. Armstrong uses the ligature oftener than the clamp, believing it safest. He had not as yet used the ecraseur. Believed the pain after ligaturing due chiefly to the fact that the ligature is not tied firmly enough. Had two cases where a second and firmer tying of the ligature completely relieved the pain.

Dr. Battey—The ligature is not obsolete; the majority of surgeons operate even now with the ligature. Agreed with Drs. Logan and Armstrong, that the pain is mostly due to the ligature not being tied tightly enough. The ligature should be good and strong. The motto in the London hospitals is, "cut skin and tie mucous membrane." They use no anæsthetic, tie the tumor stoutly, and the patients have but little if any subsequent pain. An opiate was seldom administered. Usually the tumors come away after three or four days. Tetanus is to be feared when the pain is not obviated by tying sufficiently to kill the parts. In other hospitals burning with nitric acid was a favorite treatment; but the ecraseur supplants the clamp, and is superior to the ligature; is safe and sure. He crushes the tumor slowly, and has never seen hemorrhage in any sense; barely more than enough to soil a white handkerchief. Has met with contraction of the rectum, but has never seen any trouble from it, for the parts can be readily dilated by suitable instruments, or even by the introduction of the thumbs. The cures by the ecraseur are far better than those by the ligature. The former seems to induce a better tone in the structures, and the roughness of the parts is often restored to the smoothness of a new-born babe.

Dr. Baird mentioned having seen a case in the hands of a distinguished surgeon in New York, where excessive hemorrhage followed the use of the ecraseur, and that the doctor remarked that it would be the last, as it was the first operation he had ever made with the ecraseur.

Dr. Battey could readily imagine serious hemorrhage in consequence of too rapid a crushing, but could not conceive how it was possible for it to occur when the instrument was slowly screwed down. He frequently takes thirty to forty minutes to complete the crushing. Time is not so much to be valued as a perfect result, now that we have anæsthesia to abolish all pain.

Adjourned.

